# Exploring the Competition between Proliferative and Invasive Cancer Phenotypes in a Continuous Spatial Model

**DOI:** 10.1371/journal.pone.0103191

**Published:** 2014-08-06

**Authors:** Eleftheria Tzamali, Georgios Grekas, Konstantinos Marias, Vangelis Sakkalis

**Affiliations:** Computational Medicine Laboratory, Institute of Computer Science, Foundation for Research and Technology-Hellas, Heraklion, Greece; University of California Irvine, United States of America

## Abstract

Tumor is characterized by extensive heterogeneity with respect to its microenvironment and its genetic composition. We extend a previously developed monoclonal continuous spatial model of tumor growth to account for polyclonal cell populations and investigate the interplay between a more proliferative and a more invasive phenotype under different conditions. The model simulations demonstrate a transition from the dominance of the proliferative to the dominance of the invasive phenotype resembling malignant tumor progression and show a time period where both subpopulations are abundant. As the dominant phenotype switches from proliferative to invasive, the geometry of tumor changes from a compact and almost spherical shape to a more diffusive and fingered morphology with the proliferative phenotype to be restricted in the tumor bulk and the invasive to dominate at tumor edges. Different micro-environmental conditions and different phenotypic properties can promote or inhibit invasion demonstrating their mutual importance. The model provides a computational framework to investigate tumor heterogeneity and the constant interplay between the environment and the specific characteristics of phenotypes that should be taken into account for the prediction of tumor evolution, morphology and effective treatment.

## Introduction

Tumor is characterized by extensive heterogeneity with respect to its microenvironment and its genetic composition that all play an important role in tumor progression, morphology, drug resistance and effective treatment [1]–[4]. Specifically, within tumor, well-vascularized regions providing sufficient nutrients to cancer cells coexist with nutrient-limited regions. In addition to that, there is much evidence to support intra-tumor genetic and functional heterogeneity in many cancer types [5], [6]. In gliomas, for example, the most common brain tumors, differentially expressed genes have been identified in the invading rim and the tumor core and these differences have been phenotypically mapped to two distinct sub-clones, the proliferative cells at the core and the migratory, invasive cells in the rim [7], [8]. Recently, an integrated genomic analysis [9] has also revealed extensive intra-tumor heterogeneity in glioblastomas at genotype, phenotype and molecular evolution level within the same tumor and showed that spatially distinct tumor samples display different glioblastoma subtypes. The intra-tumor phenotypic heterogeneity has been mainly attributed to increased epigenetic alternations as well as the accumulation of mutations throughout tumor development. Furthermore, it has been also suggested that primary tumors may already consist of genetically heterogeneous populations of cancer cells accommodating even highly aggressive and metastatic phenotypes from their origin [10]. These different cancer populations are in a constant interplay with each other and their microenvironment competing for space, resources and other factors. Their interactions shape the microenvironment, which in turn acts as a selective force on clonal emergence and evolution.

Several mathematical models have been proposed to describe the complex, spatiotemporal evolution of tumors [11], [12]. Among them, discrete and hybrid discrete-continuous mathematical models can incorporate various phenotypes, link genotypes with phenotypes and describe the behavior of each cancer cell individually as it interacts with its microenvironment [13]–[16]. Yet, these approaches are computationally expensive and therefore more suitable for small populations. On the other hand, continuous mathematical models are commonly used to describe the growth of large tumors focusing more on the collective, averaged behavior of tumor cells [17]–[20]. These approaches usually assume monoclonal tumor populations.

In this work, we extend a previously developed continuous spatial model of monoclonal tumor growth [17], [21] to account for two cancer subpopulations of distinct phenotypic characteristics that play an important role in tumor growth, invasion and metastasis. In particular, we assume a vascularized solid tumor that consists of one more proliferative and another more motile/invasive phenotype, which are in a constant competition for space and resources within the tumor microenvironment. The construction of the phenotypes is inspired by the proliferation-migration dichotomy mechanism [8], [22], although a larger spectrum of possible proliferation and motility rates is also investigated. Two different hypotheses are also explored regarding the invasive phenotype. i) In the first, invasion appears as a response to hypoxic conditions in accordance to experimental evidence, which supports that hypoxic stress stimulates tumor cell migration [23]–[27] and ii) in the second, the invasive behavior is adopted by a phenotype regardless of the hypoxic levels [10].

It should be noted that the proliferation-migration dichotomy has also been studied in a framework where the tumor population is assumed to exist in two different phases/states and its dynamics are governed by transition laws for exit and reentrance into these phases [28]. Under this framework, several mechanisms have been proposed to trigger the phenotypic switch in invasive tumors including random process [29], hypoxic conditions [14], local cell density [30] and cell-cell repellent mechanisms combined with cell density [16]. In our work, the tumor is assumed to consist of two different clones with distinct phenotypic properties, a more proliferative and a more motile. These phenotypes are considered fixed properties of the populations that do not change throughout tumor evolution. We explore the conditions of dominance and coexistence between the two populations and investigate their effect on tumor growth and morphology.

The model predictions demonstrate a region in the parameter space that reflects the proliferative-invasive tradeoff in the description of the different phenotypes where a transition from the dominance of a more proliferative to the dominance of a more invasive phenotype is observed resembling malignant tumor progression. The results of our work show that early tumor progression is proliferation driven. Yet, at later stage, the increasing limitation of space and resources in tumor microenvironment favors the more motile cells, as motility gives them better access to both resources and free space. However, different micro-environmental conditions and different phenotypic properties can promote or inhibit invasion. The importance of early detecting the genetic composition of an evolving tumor becomes evident.

## Materials and Methods

The mathematical model presented in this work describes the spatiotemporal evolution of tumor growth and its microenvironment using a system of coupled, partial differential equations of reaction-diffusion-haptotaxis type.

### Microenvironment

The tumor microenvironment and its genetic composition are modeled based on [17], [21]. The microenvironment consists of the extracellular matrix (ECM), *f* and the vasculature, *V*, which provides oxygen to tumor cells.

**ECM and cell movement**. In addition to their random movement, tumor cells can haptotactically migrate to denser areas of ECM [21], [31]. The structure and composition of the ECM affect cell adhesion and motility thus, play a critical role in tumor invasion, morphology and metastasis [32]. Furthermore, in order to facilitate their movement, tumor cells usually produce matrix degrading enzymes (such as Matrix Metalloproteinases) that degrade the ECM locally. Mathematical model predictions have shown [31] that when the macromolecules of the ECM are assumed homogeneously distributed, a symmetric tumor is formed despite of the genetic heterogeneity in tumor population whereas, a random distribution of the ECM might be more realistic and allows the formation of invasive tumor morphologies under specific conditions as seen in real tumors. Therefore, we assume a random distribution of the ECM (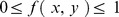), which however for simplicity is not degraded or does not change by any mechanism during tumor growth.

#### Vasculature

To minimize the modeling variables, the vasculature is assumed spatially homogeneous and temporally constant. As can be seen in (1), the oxygen (*o*) is produced by the existing vasculature at a rate *β_ο_*, diffuses at a rate *D_o_* and is consumed by normoxic and hypoxic cells at a rate *γ_οc_* and *γ_οh_*, respectively. The evolution of neovascularization is not taken into account, however by tuning the parameters *β_ο_*, *γ_οc_* and *γ_οh_*, we indirectly affect the vascularization level within the tumor.

### Oxygen-dependent cell states

The uneven balance between oxygen delivery and consumption forms regions of limited oxygen. Depending on oxygen availability cancer cells can be normoxic (*c*), hypoxic (*h*) or necrotic (*n*). As proposed in [17], normoxic cells turn to hypoxic at a rate *β* when oxygen is insufficient. Hypoxic cells can turn to normoxic at a rate *γ* if re-oxygenated, or turn to necrotic at a rate *α_h_* when oxygen becomes inadequate. Although both normoxic and hypoxic cells, when in contact with necrosis, can directly turn to necrotic, to simplify our equations, we ignore this effect. Hypoxic cells do not proliferate. The conversion rates *β* and *γ* are assumed proportional to cellular proliferation.

### Bi-clonal tumor growth model

To account for cellular heterogeneity, the population consists of two distinct phenotypes so that each normoxic sub-population (*c_1_* and *c_2_*) can be converted to its corresponding hypoxic sub-population (*h_1_* and *h_2_*) and vice versa depending on oxygen availability. Normoxic sub-populations differ with respect to their proliferation rates (*ρ_1_* and *ρ_2_*), diffusion rates (*D_c1_* and *D_c2_*) and haptotactic coefficients (*χ_c1_* and *χ_c2_*). The hypoxic sub-populations differ with each other with respect to their diffusion rates (*D_h1_* and *D_h2_*) and haptotactic coefficients (*χ_h1_* and *χ_h2_*). In general, different oxygen consumption rates and normoxic-hypoxic conversion rates could also be assumed between the different phenotypes to account for intratumoral heterogeneity. However, in order to minimize the modeling parameters and focus on those that mainly distinguish the two phenotypes, we assumed that these rates are the same for both phenotypes.

The two phenotypes are in a constant interplay competing for oxygen and space availability. The system of the coupled partial differential equations of the involved species, described in the previous paragraphs, is presented in (1) in a non-dimensionalized form. In (1), *T* corresponds to the sum of normoxic, hypoxic and necrotic cell populations. The term ‘1-*T*’ is used in these equations to reflect the inhibition of proliferation and motility due to cellular crowding and prevent the sum of tumor populations to locally exceed the tumor cell carrying capacity.
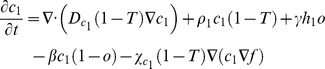


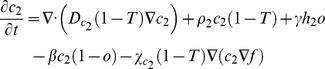


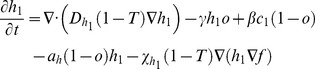
(1)

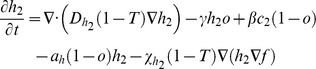


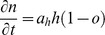


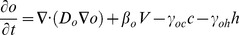
where 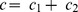, 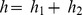 and 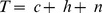
_._

The distribution of normoxic cells, 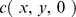, was initialized with a Gaussian function of height equal to 0.9 and standard deviation 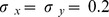 mm, resulting in an initial tumor diameter of approximately 0.6 mm (considering that tumor is detectable when cell density is above 10% of the maximum tissue carrying capacity). It is assumed that initially hypoxic and necrotic cells are not present. The oxygen concentration is initialized at its saturation value (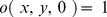). Vasculature, *V*, is constant and is assumed equal to one in the entire domain.

The non-dimensionalized form of the system of equations (1) was solved in a 800×800 grid spanning an overall area of 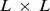, where L = 4 cm. The temporal resolution was set to *τ* = 8 h. No-flux boundary conditions are imposed for all equations. The spatiotemporal solution of the system is approximated by applying the Alternative Direction Implicit method of Finite Differences in two spatial dimensions [33], [34].

### Description of phenotypes

We assume that the initial tumor consists of one more proliferative and another more motile/invasive phenotype. We call the more proliferative phenotype, *phenotype 1* and the more motile phenotype alternatively *phenotype 2* or *phenotype 3* depending on the hypothesis, made for invasion. Specifically, Phenotype 2 is invasive under hypoxic conditions and can thus be regarded as conditionally more motile, while phenotype 3 is unconditionally more motile. Using the bi-clonal tumor growth model, the co-growth between Phenotype 1 and Phenotype 2, as well as the co-growth between Phenotype 1 and Phenotype 3 are explored under different vasculature conditions. The set of all the parameters used in our simulations are in accordance to [17], [21] and depicted in Table 1. Additionally, Table 2 shows the exact parameters used to describe each phenotype, although a range of various proliferation and motility rates is also investigated. Phenotype 1 is highly proliferative (*ρ_1_* = *ρ*), but less invasive (*D_c1_* = *D_h1_* = 0.1*D_g_*, *χ_c1_* = *χ_h1_* = 0) than phenotype 2 and phenotype 3. Phenotype 2 is less proliferative (*ρ_2_* = 0.8*ρ*) than phenotype 1 and invasive under hypoxia (*D_c2_* = 0.1*D_g_*, *D_h2_* = *D_g_, χ_c2_* = 0, *χ_h2_* = *χ*), while phenotype 3 is even less proliferative (*ρ_2_* = 0.4*ρ*) than phenotype 2, yet unconditionally invasive (*D_c2_* = *D_h2_* = *D_g_*, *χ_c2_* = *χ_h2_* = *χ*).

**Table 1:** Model parameters in dimensional and non-dimensional forms.

Parameter	Value	Nondim.
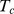	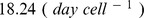	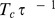
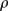	*ln2T_c_^−1^(day)^−1^*	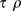
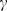	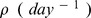	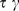
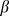	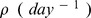	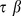
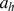	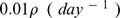	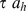
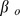	-	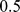
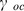	6.25⋅10^−17^ 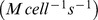	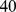
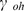	-	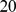
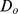	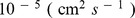	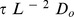
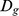	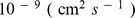	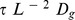
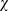	-	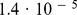

**Table 2:** Values of characteristic properties for each tumor cell phenotype.

	Proliferation (normoxic, hypoxic)	Diffusion rate (normoxic, hypoxic)	Haptotaxis (normoxic, hypoxic)	Description
**Phenotype 1**	(ρ, 0)	(0.1D_g_, 0.1D_g_)	(0, 0)	**Proliferative:** More proliferative, Non-invasive
**Phenotype 2**	(0.8ρ, 0)	(0.1D_g_, D_g_)	(0, χ)	**Invasive:** Less proliferative, Invasive under hypoxia
**Phenotype 3**	(0.4ρ, 0)	(D_g_, D_g_)	(χ, χ)	**Invasive:** Less proliferative, Unconditionally more invasive

### Intra-tumor vasculature

Tumor growth is studied under two relatively different vasculature conditions. Specifically, we assume a poorly-vascularized tumor where vasculature has not been established very effectively and a well-vascularized tumor with a relatively more effective development of vasculature within the tumor. As neovascularization has not explicitly modeled, we indirectly affect the vascularization level within tumor by varying the oxygen consumption rates. In the poorly vascularized case study, the parameters for the oxygen uptake rates are shown in Table 1. On the other hand, in order to mimic a more vascularized tumor, we reduced the oxygen consumption rates to 1/10 of their original values. Even with the established vasculature, as tumor grows in size, oxygen supply becomes inadequate and areas of hypoxia and necrosis are developed within tumor. The difference between the poor and well vascularized simulated conditions lies in the more or less rapid hypoxia onset, respectively.

## Results

### Hypoxia-induced invasive phenotype dominates highly proliferative one

In our first set of experiments, we investigate the growth of a tumor that consists of a proliferative and hypoxia-driven invasive phenotype, under both poor and well vascularized conditions. Thus, the *in-silico* tumor initially consists of phenotype 1 and phenotype 2, as outlined in *Materials and Methods* section. Phenotype 1 is assumed abundant in the initial population *c_1_*(0):*c_2_*(0)  = 0.95∶0.05 to emphasize our outcomes, although equal-sized initial populations were also simulated and produced similar results.

#### Poorly-vascularized growth conditions

**Figure 1:**
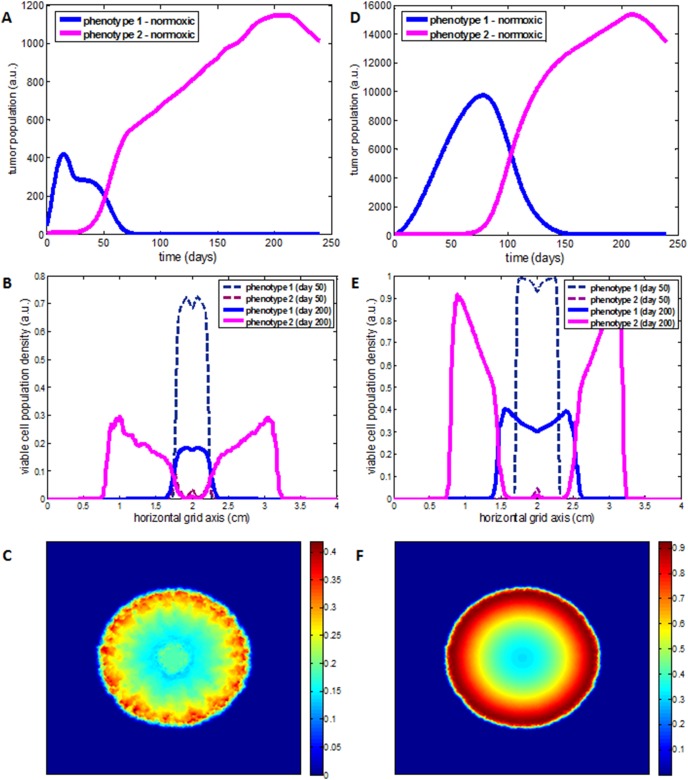
An *in-slico* tumor consisting of a proliferative (phenotype 1) and a hypoxia-induced invasive (phenotype 2) sub-population as grows under poorly-vascularized (on the left column) and well-vascularized conditions (on the right column). A, D) The evolution of the corresponding normoxic sub-populations of each phenotype illustrates the dominance of phenotype 2. B, E) A central cross section of the tumor at day 50 and day 200 showing the spatial distribution of the viable sub-populations of the two phenotypes, respectively. C, F) The spatial distribution of viable (normoxic and hypoxic) cells after 200 fictitious days varying from blue color at the lowest cell density to red color at the highest observed as depicted in the corresponding colorbar.

Figure 1A shows the evolution of the normoxic sub-population of each phenotype. Phenotype 1 initially dominates in the population, while phenotype 2 only slightly grows. However, very rapidly after the onset of hypoxia (within 50 days of simulated tumor growth) both phenotypes coexist in abundance and after this time period, phenotype 2 becomes dominant resulting in the final elimination of the normoxic sub-population of phenotype 1. It should be noted that hypoxic cells of phenotype 1 can still be found in the tumor population (Figure S1), indicating that this balance could be changed if the vascularization level within the tumor changes. In addition to the temporal evolution, the spatial distribution of the two evolving phenotypes is also interesting showing how these populations are located within the tumor. As can be seen in Figure 1B, at day 50, phenotype 1 spatially dominates over phenotype 2, while both phenotypes are widely distributed in the tumor. However, after 200 days of simulation, phenotype 1 is strictly located in the center covering an area of radius 0.35 cm approximately, while phenotype 2 has been expanded and located at tumor edges forming the invasive front of length 0.9 cm approximately.

Throughout the transition from the dominance of the more proliferative to the dominance of the more invasive phenotype, the morphology of the simulated tumor also switches from a solid and circular tumor to a highly diffusive tumor (Video S1). A snapshot of the spatial distribution of viable (normoxic and hypoxic) cells at day 200, is illustrated in Figure 1C. Due to the rapid dominance of the invasive phenotype, the density of the viable cells is relatively low. As expected, denser areas of viable cells are located at tumor edges, while morphological instabilities in the tumor front are formed, as phenotype 2 moves to local gradients of the extracellular matrix.

#### Well-vascularized growth conditions

In the well-vascularized tumor experiments, the appearance of hypoxia is significantly delayed resulting in a delayed dominance of the invasive phenotype. As can be seen in Figure 1D, while phenotype 1 initially dominates and phenotype 2 slightly grows, after about 100 days of simulated tumor growth, both phenotypes are abundant. Thereafter, phenotype 2 becomes dominant, while the population of phenotype 1 is reduced to near extinction. The progressive dominance of phenotype 2 is evident in both normoxic (Figure 1D) and hypoxic subpopulations (Figure S3). As shown in Figure 1E, the spatial distribution of the two evolving phenotypes shows that at day 200, phenotype 1 is restricted in the tumor center, which radius is 0.63 cm approximately, whereas phenotype 2 dominates at tumor edges composing the invasive front of length 0.62 cm approximately. The tumor diameter at day 200 is similar in size with the poorly-vascularized tumor (Figure S2 and Figure S4), which is approximately equal to 2.5 cm. Figure 1E also shows that under well intra-tumor vascularization, the spatial extent of phenotype 1 is larger and the density of the invasive cells in the tumor front are considerably higher than in the poorly-vascularized tumor (shown in Figure 1B), which is expected as the prolonged period with plenteous oxygen promotes cellular growth. Furthermore, the distribution of the density of the viable cells at day 200 (Figure 1F) is considerably more dense and solid compared with the poorly-vascularized tumor (Figure 1C). The evolution of the viable cells from the beginning of the simulations can be seen in Video S2.

The simulations combined show that although the proliferative phenotype initially outgrows, after the onset of hypoxia, the hypoxia-induced invasive phenotype becomes the dominant sub-population in both well- and poorly-vascularized tumors even when it is less frequent in the initial population. The simulations also show that phenotype 1 is strictly located in the center, while phenotype 2 lies at tumor edges. As expected, the onset of hypoxia is important for the outgrowth of the invasive phenotype and poor intra-tumor vascularization promotes its dominance. Additional experiments (Figure S5, Text S1) demonstrate that when phenotype 1 and phenotype 2 co-grow under normoxic conditions, phenotype 1 is favored instead, while phenotype 2 is trapped in the tumor core and its growth is stalled. Furthermore, experiments where we change the initial period of normoxia (Figure S6, Figure S7 and Text S1) also show that the exact timing of the hypoxia onset is also critical. If the normoxic period lasts long enough, the growth of phenotype 2 is stalled. Interestingly, the population dynamics can also change, if oxygen becomes plentiful after some point in time (Figure S8, Text S1).

### Normoxia accelerates the dominance of the unconditionally invasive phenotype

In this set of experiments, we consider the co-growth of a more proliferative and unconditionally more motile phenotype, under both poor and well vascularized conditions. The initial tumor thus, consists of the proliferative phenotype 1 and the invasive phenotype 3 (Table 2). Again, in all the experiments, the proliferative to invasive initial population ratio is *c_1_*(0):*c_2_*(0)  = 0.95∶0.05. However, equal-sized initial populations were also simulated and the results were similar.

#### Poorly-vascularized growth conditions

**Figure 2:**
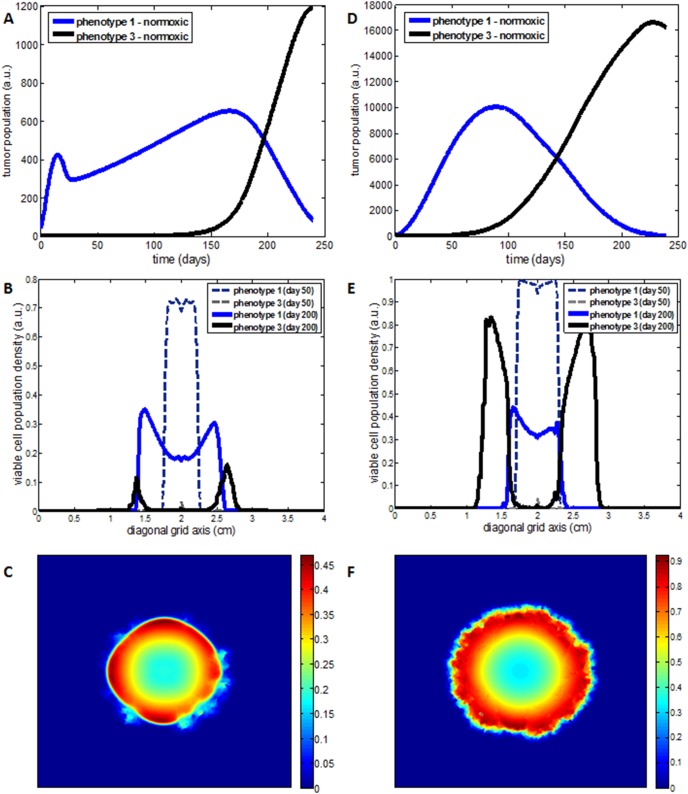
An *in-slico* tumor consisting of a highly proliferative (phenotype 1) and a highly invasive (phenotype 3) sub-population as grows under poorly-vascularized (on the left column) and well-vascularized conditions (on the right column). A, D) The evolution of the corresponding normoxic sub-populations of each phenotype illustrates the dominance of phenotype 3. B, E) A central cross section of the tumor at day 50 and day 200 showing the spatial distribution of the viable sub-populations of the two phenotypes, respectively. C, F) The spatial distribution of viable (normoxic and hypoxic) cells after 200 fictitious days varying from blue color at the lowest cell density to red color at the highest observed, as depicted in the corresponding colorbar.

The concentration of the normoxic sub-populations of each phenotype over time is shown in Figure 2A. For a very long period from the beginning of the simulations, phenotype 1 dominates within tumor, while phenotype 3 appears non-growing as if asleep. Surprisingly however, after approximately 150 days, phenotype 3 starts to rapidly grow and dominate in the population, while the population of phenotype 1 declines. It should be noted that the corresponding hypoxic sub-populations of the two phenotypes show similar dynamic behavior, yet a transition from the dominance of the proliferative to the dominance of the invasive phenotype does not occur within 240 days of tumor growth simulation (Figure S9). The spatial distribution of the two evolving phenotypes is illustrated in Figure 2B. Although the spread of the invasive phenotype at tumor edges can be seen in Figure 2B and Figure 2C, the spatial dominance of the proliferative phenotype is evident in almost the whole tumor domain. The total tumor diameter at day 200 is approximately 1.9 cm, while the invasive protrusions have a length approximately equal to 0.25 cm. Figure 2C illustrates the spatial distribution density of the viable cells at day 200, whereas its evolution from the beginning of the simulations can be seen in Video S3.

#### Well-vascularized growth conditions

Figure 2D shows the evolution of the normoxic sub-populations of the two phenotypes under well-vascularized tumor growth conditions. Similarly with the poorly oxygenated tumor, a transition in the dominant population is observed from phenotype 1 to phenotype 3 during tumor growth. However, the dominance of the invasive phenotype is observed in both normoxic (Figure 2D) and hypoxic sub-populations (Figure S11) and appears approximately 50 days earlier in the normoxic population than in the poorly vascularized tumor indicating that tumor vascularization is important to compensate for the significantly reduced proliferative capacity of the invasive phenotype. At day 200, the tumor diameter is approximately equal to 2.4 cm, contrary to the poorly-vascularized tumor, which is smaller in size of approximately 1.9 cm in diameter (Figure S10 and Figure S12). Figure 2E shows the spatial distribution of the two evolving phenotypes under well-vascularized growth conditions. At the beginning of tumor growth (fictitious day 50), phenotype 1 spatially dominates over phenotype 3, whereas both phenotypes are distributed in the whole tumor domain. However, after 200 days of growth, phenotype 1 is mainly located in the tumor center covering an area of radius 0.44 cm approximately, while phenotype 3 dominates at tumor edges forming an invasive front of length 0.49 cm approximately. As tumor progresses and the dominance of phenotype 1 switches to the dominance of phenotype 3, its geometry significantly changes from a compact and almost spherical shape to a more diffusive and fingered morphology (Video S4). Figure 2F illustrates the spatial distribution density of the viable cells at day 200.

Overall, the simulations show that when phenotype 1 and phenotype 3 co-grow, the former initially dominates but the latter eventually outgrows and becomes the dominant sub-population under both well- and poorly-vascularized tumors. The progressive dominance of phenotype 3 results in a morphology change from a spherical shape to a tumor with morphological instabilities. Compared to phenotype 1, phenotype 3 is (unconditionally) more invasive yet, with significantly reduced proliferative capacity. To compensate for its significantly reduced proliferative capacity and gain time to increase its population, a prolonged normoxic period is crucial for the faster dominance of phenotype 3. Contrary to phenotype 2, normoxia, allows the dominance of phenotype 3 and accelerates its onset (Figure S13).

### Tradeoff in proliferation and invasion rates

The next question that naturally arises, concerns whether the progressive dominance of the invasive phenotype, observed in our previous experiments, is the result of the specific cellular properties (diffusion and proliferation rates) assigned on phenotypes. Therefore, we explore the dynamics of tumor populations as we vary the proliferation rate (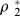) and the diffusion rate (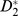) of the invasive phenotype, while keeping the rates of the proliferative, phenotype 1 constant. Furthermore, the haptotactic coefficient assigned to each newly constructed invasive phenotype has been kept the same (*χ_c2_* = *χ_h2_* = *χ*).

As we have already mentioned, the invasive phenotype can be either conditionally more invasive of type of phenotype 2 or unconditionally more invasive of type of phenotype 3. In the following experiments, we call the phenotype that is constructed when we assign different diffusion and proliferation rates from phenotype 2, phenotype 2* and phenotype 3* respectively for alterations in the rates of phenotype 3. The co-growth between phenotype 1 and phenotype 2* as well as between phenotype 1 and phenotype 3* are explored under both poor and well vascularized growth conditions. In this set of experiments, the two phenotypes under study are initially equal-sized.

**Figure 3:**
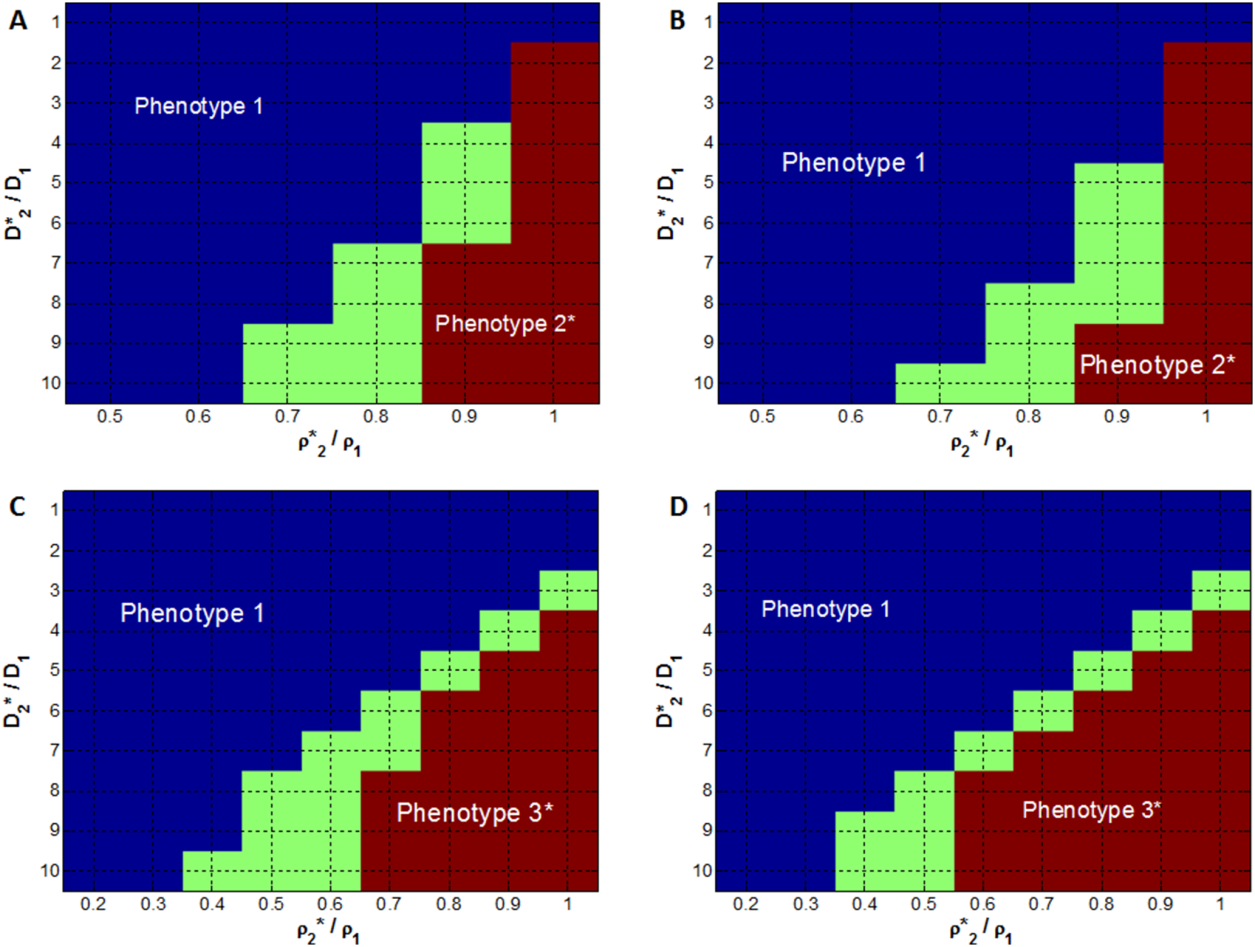
A map of the simulated tumor behavior regarding the dominant phenotypes as a function of the proliferation and diffusion rate of the invasive phenotype relative to phenotype 1 for A, B) the hypoxia-induced invasion (phenotype 2*) and C, D) the unconditional more invasive phenotype (phenotype 3*), under poor and well vascularized conditions, respectively. Points with blue color correspond to parametric pairs 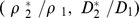 where the proliferative phenotype 1 dominates. With red color is represented the dominance of the invasive phenotype and with green color is depicted the region where a transition from the dominance of the proliferative phenotype 1 to the dominance of the invasive phenotype is observed.

Figure 3 summarizes the regions in the parameter space where each phenotype dominates for each case we just mentioned. Specifically, in the relative proliferation-invasion rate map of Figure 3, the parameter pairs 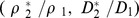 at which phenotype 1 dominates throughout tumor growth are depicted with blue color, while the dominance of the invasive phenotype, phenotype 2* or phenotype 3* is illustrated with red color. On the other hand, the green color corresponds to parameter values where a transition from the dominance of phenotype 1 to the dominance of the invasive phenotype is observed at some point throughout tumor evolution, where the two phenotypes are in a relative abundance.

Figure 3A and Figure 3C correspond to the poorly-vascularized growth conditions, while Figure 3B and Figure 3D correspond to the well-vascularized conditions. Slight differences can be observed between the two conditions. However, what is mainly observed is a difference between phenotype 2* (Figure 3A and Figure 3B) and phenotype 3* (Figure 3C and Figure 3D) with respect to the area each of them covers in the parameter space. Although phenotype 1 dominates in most areas of the parameter space explored, phenotype 3* relative to phenotype 2* covers a larger area of the parameter space indicating that an unconditionally invasive phenotype has a wider range of combinations for proliferation and diffusion rates that allows it to dominate when it co-grows with phenotype 1. Furthermore, the simulations demonstrate that a transition from the dominance of the proliferative to the dominance of the invasive phenotype is observed when the proliferation-invasion tradeoff is captured in the description of the two phenotypes; otherwise, a single phenotype dominates throughout tumor evolution. The negative relationship between proliferation and invasion is particularly evident in the unconditional invasion hypothesis (Figure 3C and Figure 3D). Interestingly, the transition in the dominance of the two phenotypes can be achieved within a narrow region of the relative invasion-proliferation space and approximated by a linear function showing that keeping the relative ratio of the growth rate to diffusion coefficient fixed, similar dynamics could be obtained. On the contrary, the proliferation-invasion tradeoff conditions are looser when invasion is hypoxia-driven, allowing thus higher variance in the potential diffusion rate of the invasive phenotype for a particular proliferation rate. Not surprisingly, the reduction in the proliferative capacity of the unconditionally invasive phenotype (phenotype 3*) can be significantly higher than that of the hypoxia-dependent invasive phenotype (phenotype 2*) because the phenotypic properties of the former allow better accessibility to vital oxygen. Further decrease in the proliferative capacity of the invasive phenotype, prevents its growth when it competes with the highly proliferative phenotype. On the other hand, a phenotype, which is both highly invasive and highly proliferative, immediately dominates over the proliferative, non-invasive phenotype. Further details and additional simulations with respect to the proliferation-diffusion rate maps can be found in Figure S15–S16 and Text S2.

## Discussion

In this work, we extend a previously developed monoclonal continuous spatial model of tumor growth [17] to account for polyclonal cell populations. As a starting point, we assume two cancer subpopulations, which form the initial tumor and have distinct phenotypic characteristics that do not change throughout tumor evolution. These different cancer populations are in a constant competition for space and resources within the tumor microenvironment.

Utilizing the proposed bi-clonal model, we specifically investigate the interplay between proliferation and invasion and explore the co-evolution of distinct phenotypes with these properties, the conditions of their dominance and coexistence under competition as well as their effect on tumor growth and morphology. In particular, we assume that the tumor consists of one more proliferative phenotype and another more motile/invasive. The construction of the phenotypes is inspired by the proliferation-migration dichotomy [8], [22], although a larger spectrum of combinations for proliferation and motility rates is also investigated. Regarding the invasive phenotype, two different hypotheses are explored. In the first, invasion appears as a response to hypoxic stress and in the second, the invasive behavior is adopted by a phenotype unconditionally. In all simulations, a vascularized solid tumor has been assumed, although vascularization has been implicitly introduced and its mathematical representation has been oversimplified. Tumor evolution is studied under poorly and well vascularized growth conditions. Both conditions develop hypoxia within the tumor, but imply a less or more rapid initiation of hypoxia, respectively.

Our model simulations demonstrate the malignant development of an initially small tumor and show that early tumor progression is proliferation driven but, as tumor grows, the increasing competition for space and resources favors the more motile cells. At the early stages of tumor growth, proliferative cells dominate and supplant invasive cells, which lying as if dormant during this period. However, as tumor grows the invasive phenotype outgrows and dominates in the population. When the proliferative cost applied on the invasive phenotype is considerably large, yet not too large, the simulations have revealed a transition from the dominance of the proliferative to the dominance of the invasive phenotype and a time period where both subpopulations are abundant. Further simulations have also shown that a decrease in the motility of the invasive cells must be followed by a proper increase in their proliferative capacity to achieve similar population dynamics. If there is not such a tradeoff, the phenotype, which is both highly invasive and highly proliferative, immediately dominates over a non-invasive phenotype. It should also be noted that a monoclonal tumor where adaptation or mutations are allowed to occur throughout tumor evolution can obtain similar transition dynamics as for example phenotype 2, which becomes invasive under hypoxia. Nevertheless, in addition to mutation effects and environmental pressure for adaptation on a phenotype, growing experimental evidences support that tumors are highly heterogeneous [2] even from their origin [10] making interactions among phenotypes and their environment inevitable.

Not surprisingly, the evolution of the invasive phenotypes depends on the oxygenation level within tumor as the escape of tumor cells from the nutrient limited core allows greater access to oxygen. In the case where the more proliferative phenotype (phenotype 1) co-grows with the hypoxia-induced invasive phenotype 2, hypoxia becomes a critical factor for the outgrowth of phenotype 2. In fact, under normoxic conditions, phenotype 2 is trapped in the tumor core and its growth is stalled. On the other hand, when phenotype 1 co-grows with the unconditionally more motile phenotype 3, we observe that normoxia actually accelerates the dominance of phenotype 3, as it allows it to compensate for its significantly reduced proliferative capacity and gain time to increase its population. Additionally, the co-growth between the different types of invasion is also interesting. Further analysis has revealed that under competition, phenotype 2, which is hypoxia-driven invasive, dominates the unconditionally invasive phenotype 3, in both well and poorly vascularized tumor growth conditions (Figure S14). Furthermore, comparing the growth evolution between monoclonal and bi-clonal tumors is also revealing (Figure S17 and Figure S18). Among monoclonal populations, phenotype 2 outperforms, under both poor and well vascularized conditions, while phenotype 1 and phenotype 3 alternate in performance depending on the conditions. In bi-clonal tumors, the growth curve of the total population follows the dynamics of the fastest phenotype, although it is evident that coexistence affects each other’s growth.

The transition from the dominance of the proliferative to the dominance of the invasive phenotype is also followed by a morphological transition resembling tumor progression to higher grades of malignancy. In particular, during the period where the proliferative phenotype dominates a compact, circular tumor is formed, while the dominance of the invasive phenotype is characterized by the formation of diffusive tumors with morphological instabilities and finger-like protrusions that appear as tumor cells move to local gradients of the extracellular matrix. Apart from the noticeable morphological differences, tumor cross-sections are also revealing as they show the spatial distribution of the sub-populations during tumor evolution. At the early period of tumor growth the proliferative phenotype spatially dominates over the invasive phenotypes. However, as tumor grows, the proliferative phenotype is mainly restricted in the tumor bulk, while the invasive phenotypes dominate at tumor edges under both well and poorly vascularized tumor conditions, an observation, which is supported experimentally by the identification of genes with significantly different expression in isolated glioma cells from the invasive edge and from the tumor core [7]. Depending on the experimental setting, the different phenotypes can be considerably more or less dispersed within tumor allowing the invasive front to range from approximately 0.4 cm to 1 cm, whereas the density of the cells comprising the invasive front might be below the threshold of detection by conventional imaging method (e.g. T2 threshold of detection is approximately 16% of the maximal tissue carrying capacity) thus, misleading diagnosis and complicating the prognosis. These observations emphasize the need for mathematical modeling and stress out the fact that multiple biopsies are required in clinical practice for more accurate prognosis, grading and therapy planning and ideally should be taken across both different time points and different spatial regions within the same tumor as also proposed in [9].

The simulations have also confirmed that the radial rate of tumor expansion is approximately constant, as has been observed in gliomas [35], [36] and predicted different constant rates for the different periods of dominance. Within 200 days of simulation time, the *in-silico* tumor has increased approximately from 0.6 mm to 2.0–2.5 cm in diameter with expansion rate, which ranges from 0.093 mm/day to 0.123 mm/day depending on the specific experimental settings. In all cases, the expansion velocity during the dominance of the more invasive phenotype was higher than the velocity estimated in the period where the more proliferative phenotype dominates (Figure S2, Figure S4, Figure S10 and Figure S12) reflecting their different kinetics.

However, it should be noted that although some general properties of tumor evolution and morphology are captured in our simulations, real tumors are highly more heterogeneous and complex and considerably less well-defined and symmetric to allow any direct comparison. In reality, oxygen is not homogeneously provided, but depends on blood vessel characteristics. Furthermore, real tumor vessels are highly chaotic, tortuous, dilated and leaky with increased variance in blood flow that lead to further heterogeneity in oxygen supply. In addition to vasculature heterogeneity, the complex structure and composition of the ECM play a critical role in tumor invasion, morphology and metastasis [32] as they affect cell adhesion and motility through mechanisms known as haptotaxis and haptokinesis. The ECM is remodeled during tumor growth and tumor cells produce matrix degrading enzymes that degrade it locally to further facilitate their movement. As already mentioned, in order to keep our model simple, we assumed a random distribution of the ECM, which is not degraded or remodeled by any mechanism but remains unchanged throughout tumor growth. It is important to mention that when ECM degradation will be included it will probably favor further the invasive phenotypes. On top of all that, we should also mention that because of the increased solid stress coming from tumor growth and ECM remodeling, additional forces act on both tumor cells and blood vessels that can further affect tumor microenvironment, tumor progression and morphology.

Taken all together, it becomes clear that the combined interplay between the environment and the specific characteristics of the phenotypes should be taken into account for the prediction of tumor evolution as different micro-environmental conditions and different phenotypic properties may promote or inhibit invasion, while it questions the efficacy of anti-cancer treatment that targets the highly proliferative cells only or the microenvironment. Nevertheless, the ability to early detect the genetic composition of an evolving tumor long before it outgrows in the population could significantly improve prognosis.

### Future directions

This study extends the modeling of tumor growth that considers monoclonal cancer cell populations to polyclonal tumors and attempts to describe the coexistence of proliferative and invasive phenotypes as both types play an important role in tumor progression, invasion and metastasis. In spite of that, the presented bi-clonal model can be used to explore different physiological tradeoffs and can be easily extended to a polyclonal model in order to describe alternative forms of phenotypic heterogeneity within a tumor. A variety of different phenotypic characteristics and metabolic capabilities such as different oxygen consumption rates, proliferation rates and growth dependences on additional vital nutrients could also be assumed between the subpopulations to account for intra-tumor heterogeneity.

As a first step, we have made many simplifications in the description of the model with the aim to minimize the complexity, assumptions and modeling parameters, while we focus on those that mainly characterize the two phenotypes, affect their evolution and describe their competitive interactions with the tumor microenvironment. These simplifications include the predetermined, non-evolving properties of phenotypes, the static density of the extracellular matrix, and the oversimplified mathematical representation of the cell-matrix interactions, the vasculature and oxygen dynamics. A more realistic adaptation though can include the angiogenic cascade presented in [17], [37] and the dynamic deformation of the extracellular matrix as described in [31]. In addition, it has been reported that cancer cells not only compete for space and resources by communicating with their micro-environment, but also communicate with each other [4] through, for example, autocrine and paracrine signaling [15], [38]. Such an intra-population communication plays an important role in tumor evolution that could be incorporated in future simulations.

## Supporting Information

Figure S1 **Evolution of an in-silico tumor consisting of phenotype 1 and phenotype 2 growing under poorly-vascularized conditions.** a) The evolution of the normoxic sub-populations for each phenotype shows the final dominance of phenotype 2. b) The evolution of the corresponding hypoxic sub-populations of each phenotype showing the dominance of phenotype 2. c) A central cross section of the tumor at day 239 showing the spatial distribution of the viable sub-populations of the two phenotypes. d) A central cross section of the tumor at day 239 showing the spatial distribution of normoxic, hypoxic and necrotic cells. e) The spatial distribution of normoxic cells after 239 fictitious days. f) The spatial distribution of viable (normoxic and hypoxic) cells after 239 fictitious days.
(TIF)

Figure S2 **Evolution of tumor diameter and oxygenation levels in an in-silico tumor consisting of phenotype 1 and phenotype 2 growing under poorly-vascularized conditions.** a) Tumor diameter over time (blue line) and its linear approximation (dotted red line) b) Tumor diameter over time (blue line) as approximated by different linear functions for the different periods of dominance. During the first 50 days of growth simulations where phenotype 1 dominates in the population (dotted red line), the radial velocity of expansion is approximately equal to 0.088 mm/day whereas during the dominance of phenotype 3 (dotted green line), the tumor velocity is increased to 0.125 mm/day. c) The evolution of the minimum, maximum and total normalized oxygen level in the whole spatial domain.
(TIF)

Figure S3 **Evolution of an in-silico tumor consisting of phenotype 1 and phenotype 2 growing under well-vascularized conditions.** a) The evolution of the normoxic sub-populations for each phenotype shows the final dominance of phenotype 2. b) The evolution of the corresponding hypoxic sub-populations of each phenotype showing the dominance of phenotype 2. c) A central cross section of the tumor at day 239 showing the spatial distribution of the viable sub-populations of the two phenotypes. d) A central cross section of the tumor at day 239 showing the spatial distribution of normoxic, hypoxic and necrotic cells. e) The spatial distribution of normoxic cells after 239 fictitious days. f) The spatial distribution of viable (normoxic and hypoxic) cells after 239 fictitious days.
(TIF)

Figure S4 **Evolution of tumor diameter and oxygenation levels in an in-silico tumor consisting of phenotype 1 and phenotype 2 growing under well-vascularized conditions.** a) Tumor diameter over time (blue line) and its linear approximation (dotted red line). b) Tumor diameter over time (blue line) as approximated by different linear functions for the different periods of dominance. During the first 85 days of growth simulations where phenotype 1 dominates in the population the radial velocity of expansion is approximately equal to 0.109 mm/day (dotted red line), whereas during the dominance of phenotype 3, the tumor velocity is increased to 0.129 mm/day (dotted green line). c) The evolution of the minimum, maximum and total normalized oxygen level in the whole spatial domain.
(TIF)

Figure S5 **In-silico tumor progression consisting of phenotype 1 and phenotype 2 growing under ideal oxygen conditions (normoxia).** (Left) The evolution of each phenotype as tumor grows shows the dominance of phenotype 1 (blue line). Very rapidly, the growth of phenotype 2 is stalled (magenta line). (Right) A central cross-section of tumor populations at day 200, shows the spatial dominance of phenotype 1 (blue line) over phenotype 2 (magenta line).
(TIF)

Figure S6 **Effect of oxygen manipulation in an in-silico tumor growth experiment consisting of phenotype 1 and phenotype 2.** a, b) Tumor growth when oxygen is kept at its maximum value for 40 and c, d) 100 days, respectively and then the system evolves under well-vascularized conditions. The evolution of the normoxic (a, c) and hypoxic (b, d) sub-population of each phenotype is shown for each scenario respectively. Phenotype 2 is trapped in the tumor core under conditions where the onset of hypoxia substantially delays.
(TIF)

Figure S7 **Effect of oxygen manipulation in an in-silico tumor growth experiment consisting of phenotype 1 and phenotype 2.** a, b) Tumor growth when oxygen is kept at its maximum value for 40 and c, d) 100 days, respectively and then the system evolves under poor-vascularized conditions. The evolution of the normoxic (a, c) and hypoxic (b, d) sub-population of each phenotype is shown for each scenario respectively.
(TIF)

Figure S8 **Effect of oxygen manipulation in an in-silico tumor growth experiment consisting of phenotype 1 and phenotype 2.** The tumor evolves for i) 60, ii) 80 and ii) 100 days, under well-vascularized conditions. After that initial time period, we reinitialize oxygen at its maximum value and we keep it at maximum thereafter. The evolution of the normoxic (left) and hypoxic (right) sub-population of each phenotype is shown.
(TIF)

Figure S9 **Evolution of an in-silico tumor consisting of phenotype 1 and phenotype 3 growing under poorly-vascularized conditions.** a) The evolution of the normoxic sub-populations for each phenotype shows the final dominance of (the normoxic population of) phenotype 3. b) The evolution of the corresponding hypoxic sub-populations of each phenotype shows the dominance of phenotype 1 and the initiation of the growth of phenotype 3 after a long period of dormancy. c) A central cross section of the tumor at day 239 showing the spatial distribution of the viable sub-populations of the two phenotypes. d) A central cross section of the tumor at day 239 showing the spatial distribution of normoxic, hypoxic and necrotic cells. e) The spatial distribution of normoxic cells after 239 fictitious days. f) The spatial distribution of viable (normoxic and hypoxic) cells after 239 fictitious days.
(TIF)

Figure S10 **Evolution of tumor diameter and oxygenation levels in an in-silico tumor consisting of phenotype 1 and phenotype 3 growing under poorly-vascularized conditions.** a) Tumor diameter over time (blue line) and its linear approximation (dotted red line). b) Tumor diameter over time (blue line) as approximated by different linear functions for the different periods of dominance. During the dominance of phenotype 1 (first 180 days of growth), the radial velocity of expansion is approximately equal to 0.086 mm/day (dotted red line) whereas, during the dominance of phenotype 3, the tumor velocity is increased to 0.135 mm/day (dotted green line). c) The evolution of the minimum, maximum and total normalized oxygen level in the whole spatial domain.
(TIF)

Figure S11 **Evolution of an in-silico tumor consisting of phenotype 1 and phenotype 3 growing under well-vascularized conditions.** a) The evolution of the normoxic sub-populations for each phenotype shows the final dominance of phenotype 3. b) The evolution of the corresponding hypoxic sub-populations of each phenotype showing the dominance of phenotype 3. c) A central cross section of the tumor at day 239 showing the spatial distribution of the viable sub-populations of the two phenotypes. d) A central cross section of the tumor at day 239 showing the spatial distribution of normoxic, hypoxic and necrotic cells. e) The spatial distribution of normoxic cells after 239 fictitious days. f) The spatial distribution of viable (normoxic and hypoxic) cells after 239 fictitious days.
(TIF)

Figure S12 **Evolution of tumor diameter and oxygenation levels in an in-silico tumor consisting of phenotype 1 and phenotype 3 growing under well-vascularized conditions.** a) Tumor diameter over time (blue line) and its linear approximation (dotted red line). b) Tumor diameter over time (blue line) as approximated by different linear functions for the different periods of dominance. During the dominance of phenotype 1 (first 120 days of growth), the radial velocity of expansion is approximately equal to 0.107 mm/day (dotted red line) whereas, during the dominance of phenotype 3, the tumor velocity is slightly increased to 0.127 mm/day (dotted green line). c) The evolution of the minimum, maximum and total normalized oxygen level in the whole spatial domain.
(TIF)

Figure S13 **In-silico tumor consisting of phenotype 1 and phenotype 3 growing under ideal oxygen conditions (normoxia).** (Left) The evolution of each phenotype as tumor grows shows the initial dominance of phenotype 1 (blue line) and the transition to the dominance of phenotype 3 (black line). (Right) A central cross-section of tumor populations at day 200 shows the spatial dominance of phenotype 1 in the tumor center (blue line) and the dominance of phenotype 3 at tumor edges (black line).
(TIF)

Figure S14 **Evolution of an in-silico tumor consisting of phenotype 2 and phenotype 3.** The evolution of the normoxic (a, c) and hypoxic (b, d) sub-populations for each phenotype shows the dominance of phenotype 3 under poor and well vascularized growth conditions, respectively.
(TIF)

Figure S15 **Indicative examples showing the co-growth between phenotype 1 and a phenotype 3 with different diffusion and proliferation rates under well-vascularized conditions.** The evolution of (a) normoxic and (b) hypoxic sub-populations for each phenotype is shown, where the properties of phenotype 3* correspond to *ρ_2_* = 0.8*ρ, D_c2_* = *D_h2_* = 0.5*D_g_* and *χ_c2_* = *χ_h2_* = *χ*. The evolution of (c) normoxic and (d) hypoxic sub-populations for each phenotype is shown, where the properties of phenotype 3** correspond to *ρ_2_* = 0.6*ρ, D_c2_* = *D_h2_* = 0.7*D_g_* and *χ_c2_* = *χ_h2_* = *χ*.
(TIF)

Figure S16
**Diffusion rate – Proliferation rate map.** A map of the simulated tumor behavior regarding the dominant phenotypes as a function of the net proliferation rate and the net invasion rate of the invasive phenotype relative to phenotype 1 for (left column) the hypoxia-induced invasion (phenotype 2*) and (right column) the unconditionally more invasive phenotype (phenotype 3*), when haptotaxis is considered in the movement of invasive cells (top row) and when it is not (bottom row). Points with blue color correspond to parametric pairs 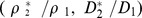 where the proliferative phenotype 1 dominates. With red color is represented the dominance of the invasive phenotype and with green color is depicted the region where a transition from the dominance of the proliferative phenotype 1 to the dominance of the invasive phenotype is observed.(TIF)

Figure S17 **Monoclonal vs. bi-clonal tumor growth.** Growth curve comparisons between the individual growth of each phenotype and their pairwise co-growths, under poor (Left) and well (Right) vascularized growth conditions, respectively. In each case, the co-growth curve is estimated by the summation of the viable cells of each phenotype (i.e. *c1+h1+c2+h2*).
(TIF)

Figure S18 **Monoclonal vs. bi-clonal tumor growth.** (Left) The evolution of the viable cells of phenotype 1, when phenotype 1 grows alone (solid blue line) and when co-grows with phenotype 3 (dashed blue line), under well-vascularized conditions. Contrary to its individual growth, the growth of phenotype 1 when co-grows with phenotype 3 decreases after a period of time. (Right) The evolution of the viable cells of phenotype 3, when phenotype 3 grows alone (solid black line) and when co-grows with phenotype 1 (dashed black line), under well-vascularized conditions. In comparison to its individual growth, the outgrowth of phenotype 3 is substantially delayed when it co-grows with phenotype 1.
(TIF)

Text S1 **A more detailed description of oxygen manipulation experiments.**
(DOCX)

Text S2 **Exploring additional changes in the diffusion-proliferation rate map.**
(DOCX)

Video S1 **Evolution of an in-silico tumor consisting of phenotype 1 and phenotype 2 growing under poorly-vascularized conditions.**
(AVI)

Video S2 **Evolution of an in-silico tumor consisting of phenotype 1 and phenotype 2 growing under well-vascularized conditions.**
(AVI)

Video S3 **Evolution of an in-silico tumor consisting of phenotype 1 and phenotype 3 growing under poorly-vascularized conditions.**
(AVI)

Video S4 **Evolution of an in-silico tumor consisting of phenotype 1 and phenotype 3 growing under well-vascularized conditions.**
(AVI)
