# Supplementary figures and images for: Exploring the Competition between Proliferative and Invasive Cancer Phenotypes in a Continuous Spatial Model
Source: PLoS One. 2014 Aug 6;9(8):e103191. doi: 10.1371/journal.pone.0103191 (PMC4123877; doi:10.1371/journal.pone.0103191)

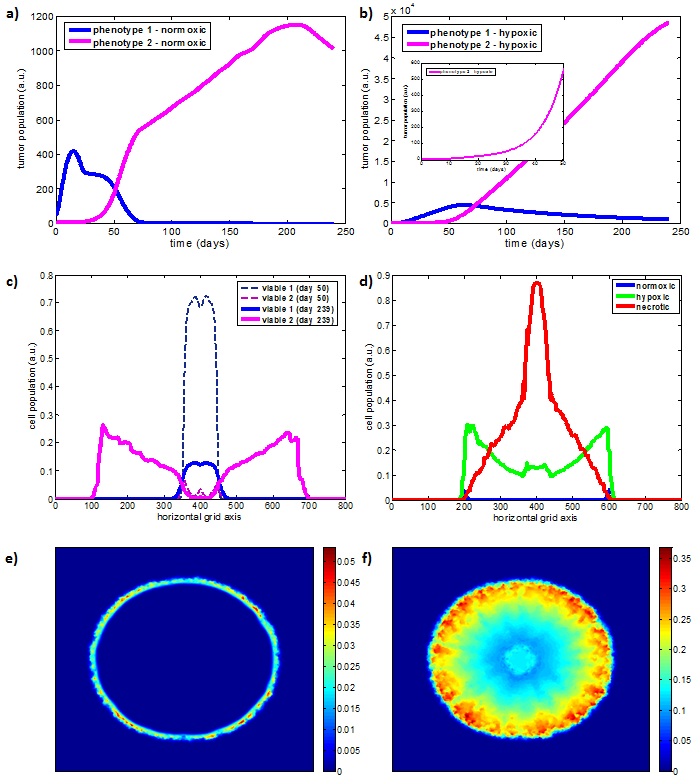

Supplement: Figure S1 — Evolution of an in-silico tumor consisting of phenotype 1 and phenotype 2 growing under poorly-vascularized conditions. a) The evolution of the normoxic sub-populations for each phenotype shows the final dominance of phenotype 2. b) The evolution of the corresponding hypoxic sub-populations of each phenotype showing the dominance of phenotype 2. c) A central cross section of the tumor at day 239 showing the spatial distribution of the viable sub-populations of the two phenotypes. d) A central cross section of the tumor at day 239 showing the spatial distribution of normoxic, hypoxic and necrotic cells. e) The spatial distribution of normoxic cells after 239 fictitious days. f) The spatial distribution of viable (normoxic and hypoxic) cells after 239 fictitious days. (TIF) [file pone.0103191.s001.tif]

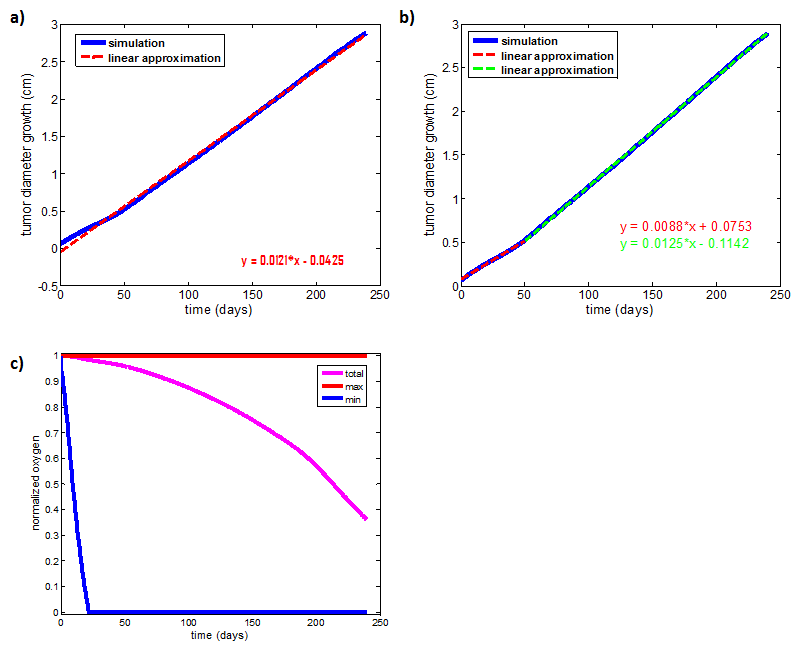

Supplement: Figure S2 — Evolution of tumor diameter and oxygenation levels in an in-silico tumor consisting of phenotype 1 and phenotype 2 growing under poorly-vascularized conditions. a) Tumor diameter over time (blue line) and its linear approximation (dotted red line) b) Tumor diameter over time (blue line) as approximated by different linear functions for the different periods of dominance. During the first 50 days of growth simulations where phenotype 1 dominates in the population (dotted red line), the radial velocity of expansion is approximately equal to 0.088 mm/day whereas during the dominance of phenotype 3 (dotted green line), the tumor velocity is increased to 0.125 mm/day. c) The evolution of the minimum, maximum and total normalized oxygen level in the whole spatial domain. (TIF) [file pone.0103191.s002.tif]

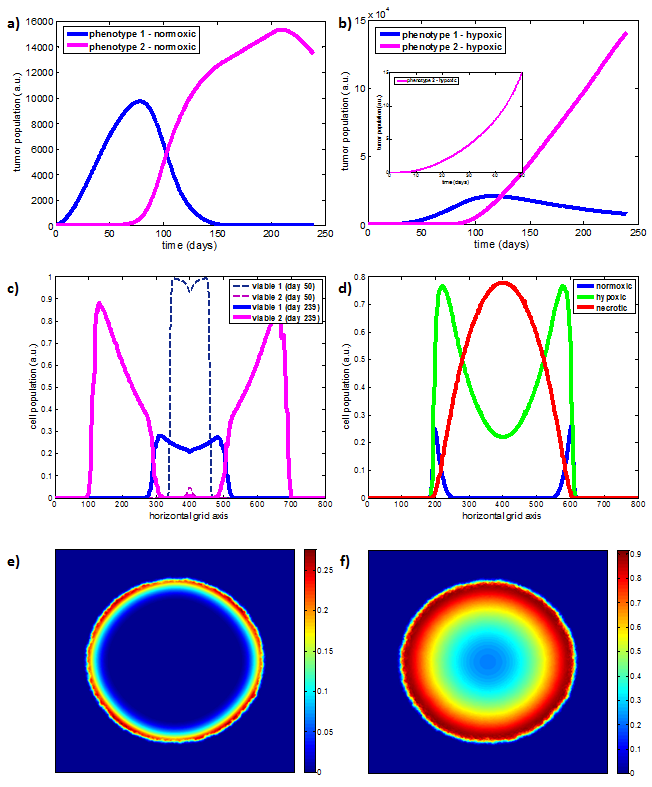

Supplement: Figure S3 — Evolution of an in-silico tumor consisting of phenotype 1 and phenotype 2 growing under well-vascularized conditions. a) The evolution of the normoxic sub-populations for each phenotype shows the final dominance of phenotype 2. b) The evolution of the corresponding hypoxic sub-populations of each phenotype showing the dominance of phenotype 2. c) A central cross section of the tumor at day 239 showing the spatial distribution of the viable sub-populations of the two phenotypes. d) A central cross section of the tumor at day 239 showing the spatial distribution of normoxic, hypoxic and necrotic cells. e) The spatial distribution of normoxic cells after 239 fictitious days. f) The spatial distribution of viable (normoxic and hypoxic) cells after 239 fictitious days. (TIF) [file pone.0103191.s003.tif]

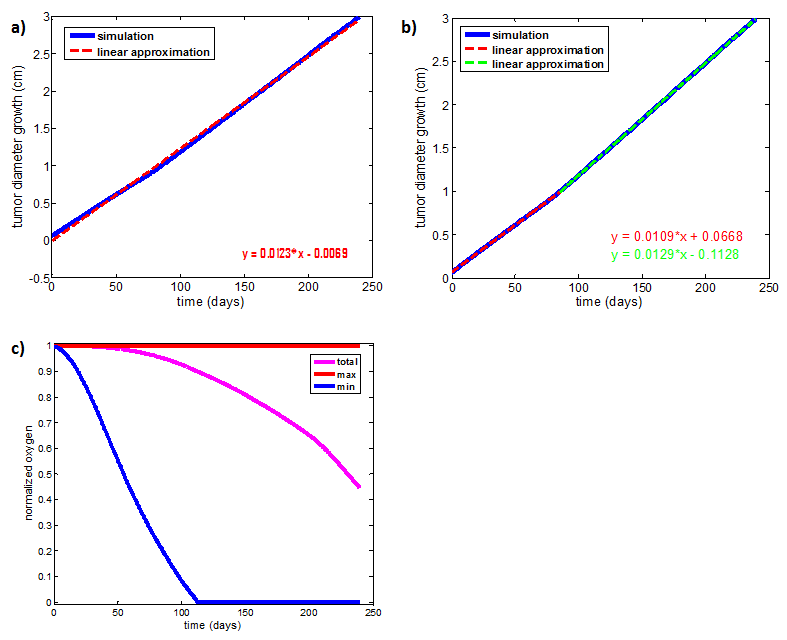

Supplement: Figure S4 — Evolution of tumor diameter and oxygenation levels in an in-silico tumor consisting of phenotype 1 and phenotype 2 growing under well-vascularized conditions. a) Tumor diameter over time (blue line) and its linear approximation (dotted red line). b) Tumor diameter over time (blue line) as approximated by different linear functions for the different periods of dominance. During the first 85 days of growth simulations where phenotype 1 dominates in the population the radial velocity of expansion is approximately equal to 0.109 mm/day (dotted red line), whereas during the dominance of phenotype 3, the tumor velocity is increased to 0.129 mm/day (dotted green line). c) The evolution of the minimum, maximum and total normalized oxygen level in the whole spatial domain. (TIF) [file pone.0103191.s004.tif]

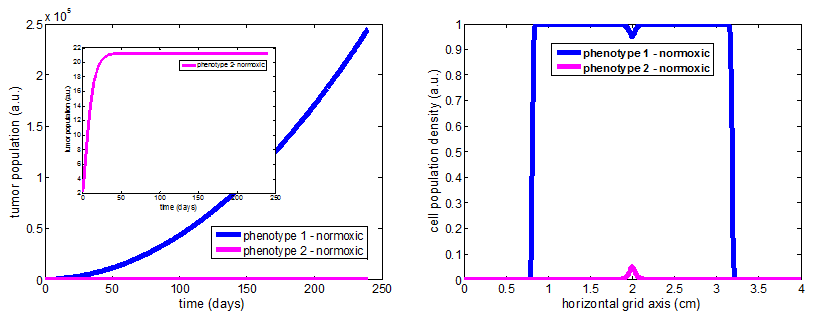

Supplement: Figure S5 — In-silico tumor progression consisting of phenotype 1 and phenotype 2 growing under ideal oxygen conditions (normoxia). (Left) The evolution of each phenotype as tumor grows shows the dominance of phenotype 1 (blue line). Very rapidly, the growth of phenotype 2 is stalled (magenta line). (Right) A central cross-section of tumor populations at day 200, shows the spatial dominance of phenotype 1 (blue line) over phenotype 2 (magenta line). (TIF) [file pone.0103191.s005.tif]

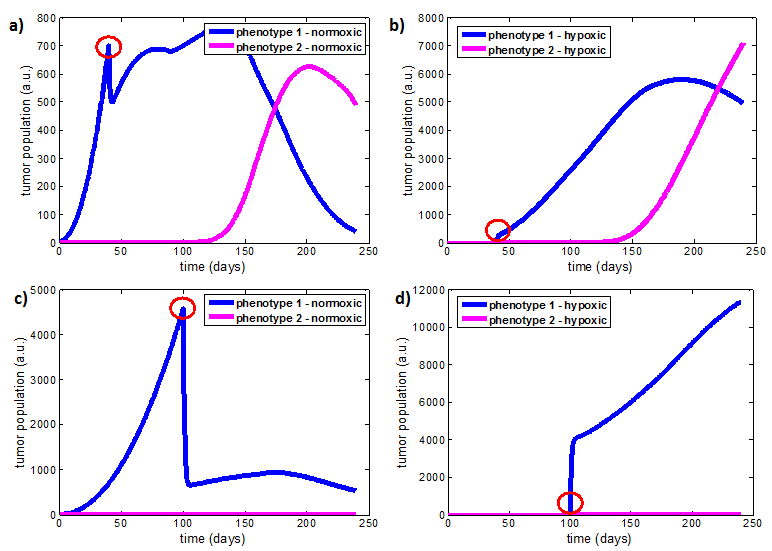

Supplement: Figure S6 — Effect of oxygen manipulation in an in-silico tumor growth experiment consisting of phenotype 1 and phenotype 2. a, b) Tumor growth when oxygen is kept at its maximum value for 40 and c, d) 100 days, respectively and then the system evolves under well-vascularized conditions. The evolution of the normoxic (a, c) and hypoxic (b, d) sub-population of each phenotype is shown for each scenario respectively. Phenotype 2 is trapped in the tumor core under conditions where the onset of hypoxia substantially delays. (TIF) [file pone.0103191.s006.tif]

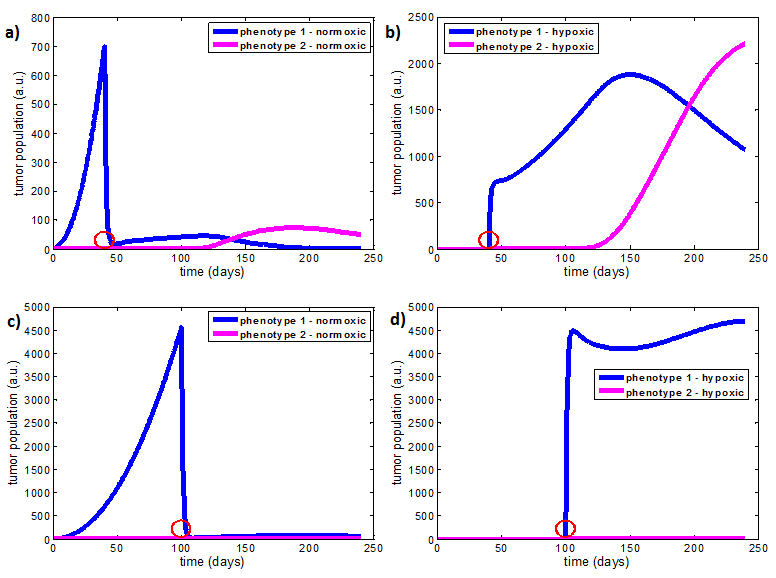

Supplement: Figure S7 — Effect of oxygen manipulation in an in-silico tumor growth experiment consisting of phenotype 1 and phenotype 2. a, b) Tumor growth when oxygen is kept at its maximum value for 40 and c, d) 100 days, respectively and then the system evolves under poor-vascularized conditions. The evolution of the normoxic (a, c) and hypoxic (b, d) sub-population of each phenotype is shown for each scenario respectively. (TIF) [file pone.0103191.s007.tif]

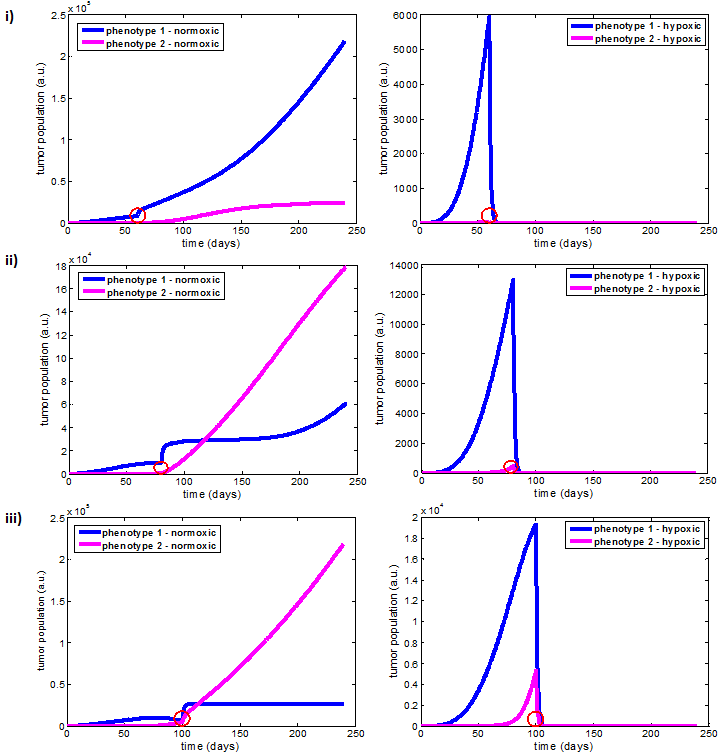

Supplement: Figure S8 — Effect of oxygen manipulation in an in-silico tumor growth experiment consisting of phenotype 1 and phenotype 2. The tumor evolves for i) 60, ii) 80 and ii) 100 days, under well-vascularized conditions. After that initial time period, we reinitialize oxygen at its maximum value and we keep it at maximum thereafter. The evolution of the normoxic (left) and hypoxic (right) sub-population of each phenotype is shown. (TIF) [file pone.0103191.s008.tif]

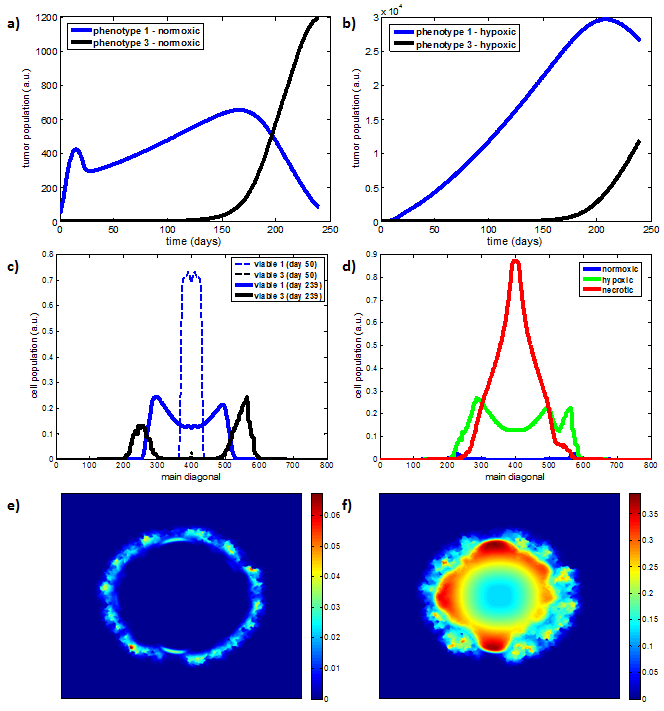

Supplement: Figure S9 — Evolution of an in-silico tumor consisting of phenotype 1 and phenotype 3 growing under poorly-vascularized conditions. a) The evolution of the normoxic sub-populations for each phenotype shows the final dominance of (the normoxic population of) phenotype 3. b) The evolution of the corresponding hypoxic sub-populations of each phenotype shows the dominance of phenotype 1 and the initiation of the growth of phenotype 3 after a long period of dormancy. c) A central cross section of the tumor at day 239 showing the spatial distribution of the viable sub-populations of the two phenotypes. d) A central cross section of the tumor at day 239 showing the spatial distribution of normoxic, hypoxic and necrotic cells. e) The spatial distribution of normoxic cells after 239 fictitious days. f) The spatial distribution of viable (normoxic and hypoxic) cells after 239 fictitious days. (TIF) [file pone.0103191.s009.tif]

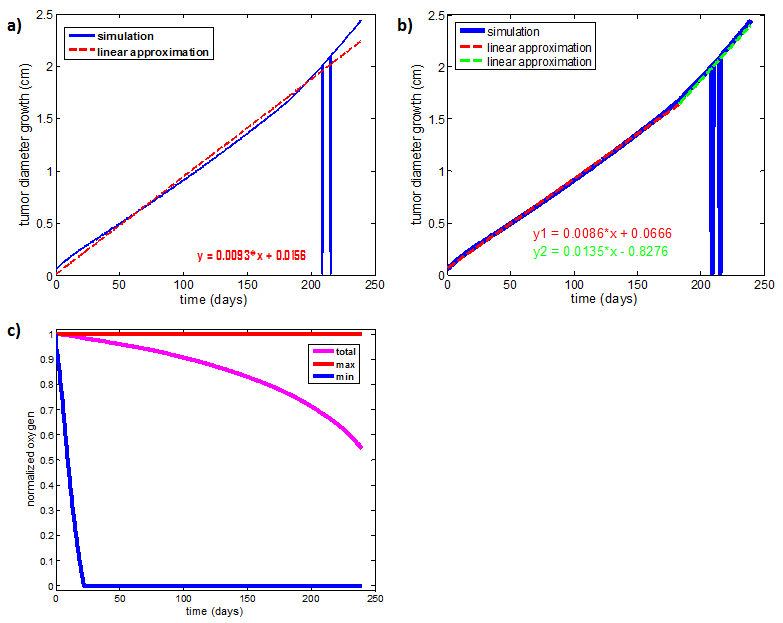

Supplement: Figure S10 — Evolution of tumor diameter and oxygenation levels in an in-silico tumor consisting of phenotype 1 and phenotype 3 growing under poorly-vascularized conditions. a) Tumor diameter over time (blue line) and its linear approximation (dotted red line). b) Tumor diameter over time (blue line) as approximated by different linear functions for the different periods of dominance. During the dominance of phenotype 1 (first 180 days of growth), the radial velocity of expansion is approximately equal to 0.086 mm/day (dotted red line) whereas, during the dominance of phenotype 3, the tumor velocity is increased to 0.135 mm/day (dotted green line). c) The evolution of the minimum, maximum and total normalized oxygen level in the whole spatial domain. (TIF) [file pone.0103191.s010.tif]

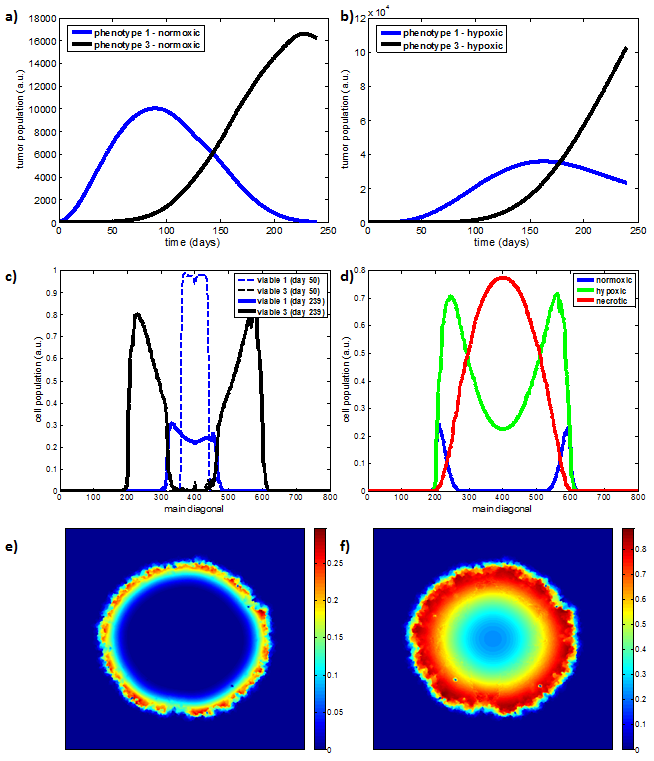

Supplement: Figure S11 — Evolution of an in-silico tumor consisting of phenotype 1 and phenotype 3 growing under well-vascularized conditions. a) The evolution of the normoxic sub-populations for each phenotype shows the final dominance of phenotype 3. b) The evolution of the corresponding hypoxic sub-populations of each phenotype showing the dominance of phenotype 3. c) A central cross section of the tumor at day 239 showing the spatial distribution of the viable sub-populations of the two phenotypes. d) A central cross section of the tumor at day 239 showing the spatial distribution of normoxic, hypoxic and necrotic cells. e) The spatial distribution of normoxic cells after 239 fictitious days. f) The spatial distribution of viable (normoxic and hypoxic) cells after 239 fictitious days. (TIF) [file pone.0103191.s011.tif]

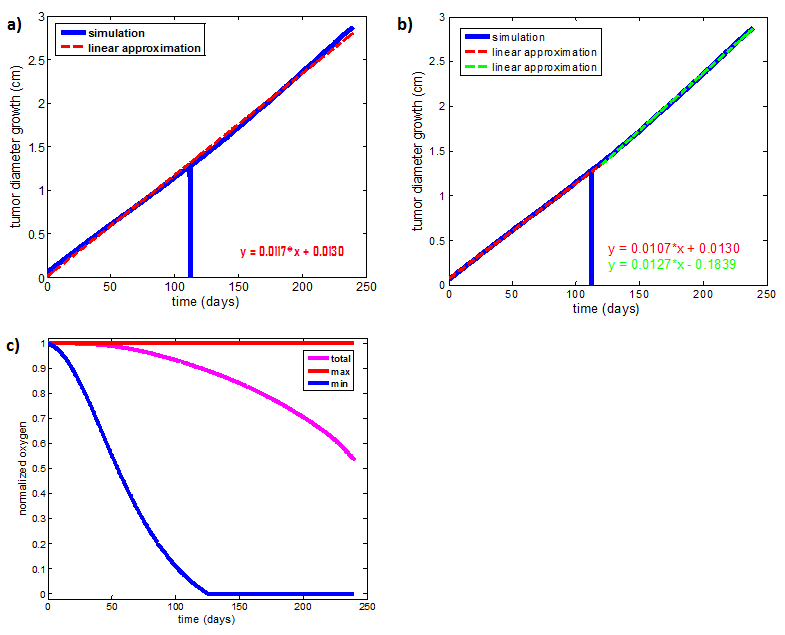

Supplement: Figure S12 — Evolution of tumor diameter and oxygenation levels in an in-silico tumor consisting of phenotype 1 and phenotype 3 growing under well-vascularized conditions. a) Tumor diameter over time (blue line) and its linear approximation (dotted red line). b) Tumor diameter over time (blue line) as approximated by different linear functions for the different periods of dominance. During the dominance of phenotype 1 (first 120 days of growth), the radial velocity of expansion is approximately equal to 0.107 mm/day (dotted red line) whereas, during the dominance of phenotype 3, the tumor velocity is slightly increased to 0.127 mm/day (dotted green line). c) The evolution of the minimum, maximum and total normalized oxygen level in the whole spatial domain. (TIF) [file pone.0103191.s012.tif]

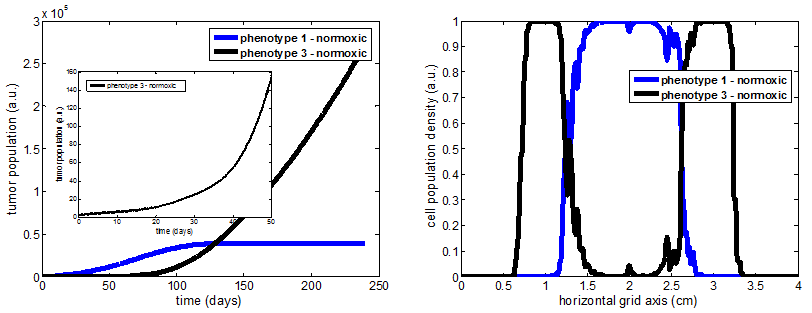

Supplement: Figure S13 — In-silico tumor consisting of phenotype 1 and phenotype 3 growing under ideal oxygen conditions (normoxia). (Left) The evolution of each phenotype as tumor grows shows the initial dominance of phenotype 1 (blue line) and the transition to the dominance of phenotype 3 (black line). (Right) A central cross-section of tumor populations at day 200 shows the spatial dominance of phenotype 1 in the tumor center (blue line) and the dominance of phenotype 3 at tumor edges (black line). (TIF) [file pone.0103191.s013.tif]

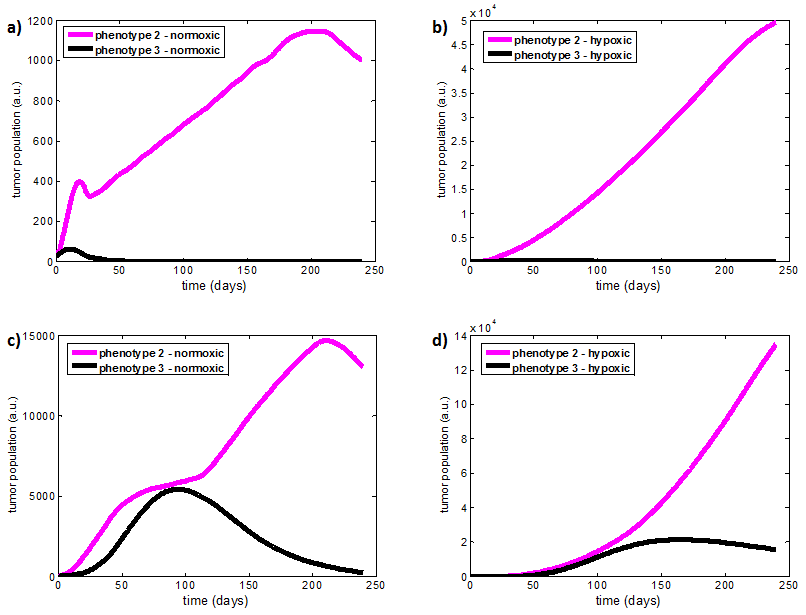

Supplement: Figure S14 — Evolution of an in-silico tumor consisting of phenotype 2 and phenotype 3. The evolution of the normoxic (a, c) and hypoxic (b, d) sub-populations for each phenotype shows the dominance of phenotype 3 under poor and well vascularized growth conditions, respectively. (TIF) [file pone.0103191.s014.tif]

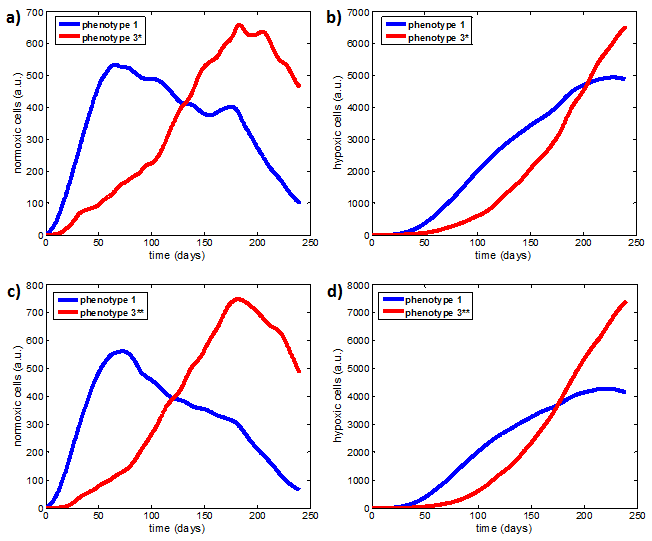

Supplement: Figure S15 — Indicative examples showing the co-growth between phenotype 1 and a phenotype 3 with different diffusion and proliferation rates under well-vascularized conditions. The evolution of (a) normoxic and (b) hypoxic sub-populations for each phenotype is shown, where the properties of phenotype 3* correspond to ρ2 = 0.8ρ, Dc2 = Dh2 = 0.5Dg and χc2 = χh2 = χ. The evolution of (c) normoxic and (d) hypoxic sub-populations for each phenotype is shown, where the properties of phenotype 3** correspond to ρ2 = 0.6ρ, Dc2 = Dh2 = 0.7Dg and χc2 = χh2 = χ. (TIF) [file pone.0103191.s015.tif]

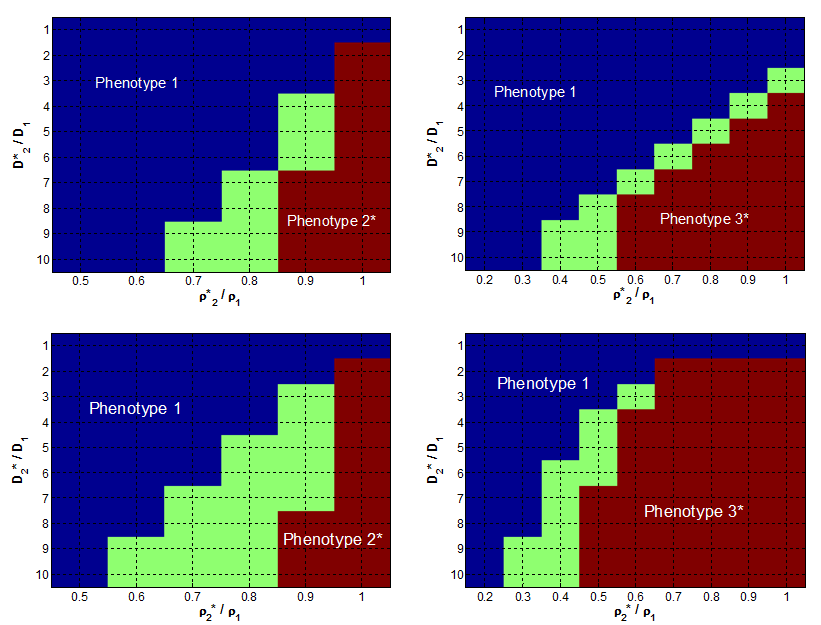

Supplement: Figure S16 — Diffusion rate – Proliferation rate map. A map of the simulated tumor behavior regarding the dominant phenotypes as a function of the net proliferation rate and the net invasion rate of the invasive phenotype relative to phenotype 1 for (left column) the hypoxia-induced invasion (phenotype 2*) and (right column) the unconditionally more invasive phenotype (phenotype 3*), when haptotaxis is considered in the movement of invasive cells (top row) and when it is not (bottom row). Points with blue color correspond to parametric pairs where the proliferative phenotype 1 dominates. With red color is represented the dominance of the invasive phenotype and with green color is depicted the region where a transition from the dominance of the proliferative phenotype 1 to the dominance of the invasive phenotype is observed. (TIF) [file pone.0103191.s016.tif]

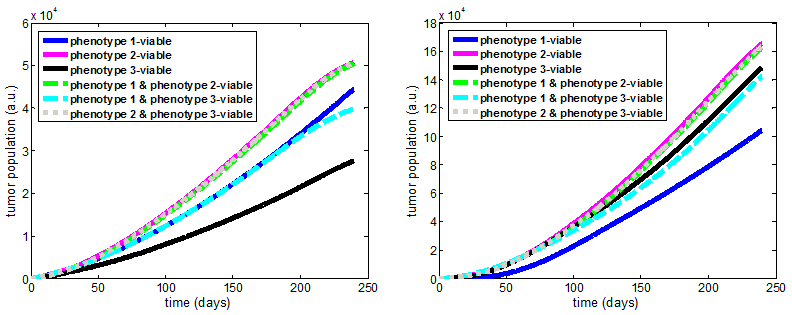

Supplement: Figure S17 — Monoclonal vs. bi-clonal tumor growth. Growth curve comparisons between the individual growth of each phenotype and their pairwise co-growths, under poor (Left) and well (Right) vascularized growth conditions, respectively. In each case, the co-growth curve is estimated by the summation of the viable cells of each phenotype (i.e. c1+h1+c2+h2). (TIF) [file pone.0103191.s017.tif]

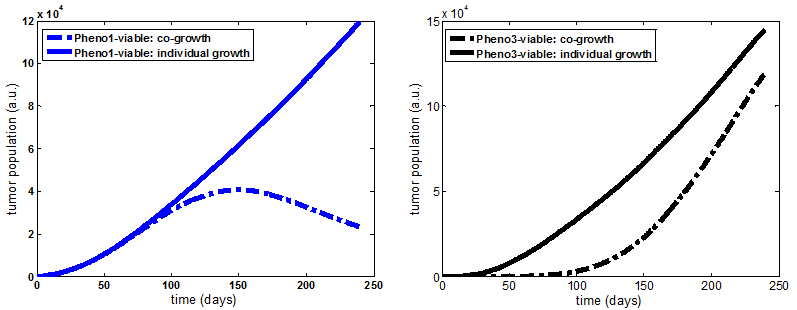

Supplement: Figure S18 — Monoclonal vs. bi-clonal tumor growth. (Left) The evolution of the viable cells of phenotype 1, when phenotype 1 grows alone (solid blue line) and when co-grows with phenotype 3 (dashed blue line), under well-vascularized conditions. Contrary to its individual growth, the growth of phenotype 1 when co-grows with phenotype 3 decreases after a period of time. (Right) The evolution of the viable cells of phenotype 3, when phenotype 3 grows alone (solid black line) and when co-grows with phenotype 1 (dashed black line), under well-vascularized conditions. In comparison to its individual growth, the outgrowth of phenotype 3 is substantially delayed when it co-grows with phenotype 1. (TIF) [file pone.0103191.s018.tif]
