# Supplementary material for: Exploring the Competition between Proliferative and Invasive Cancer Phenotypes in a Continuous Spatial Model
Source: PLoS One. 2014 Aug 6;9(8):e103191. doi: 10.1371/journal.pone.0103191 (PMC4123877; doi:10.1371/journal.pone.0103191)
Supplement: Text S1 — A more detailed description of oxygen manipulation experiments. (DOCX) [file pone.0103191.s019.docx]

**Oxygen manipulation**

We study the co-growth of phenotype 1 and phenotype 2 under various oxygen conditions.

1. Normoxic tumor

In these set of experiments, we assume that throughout tumor evolution, oxygen is always at its maximum level (*o*=1). As hypoxia and necrosis are only dependent on oxygen levels in our model, these sub-populations do not evolve under the ideal oxygen conditions. Furthermore, given that resources are never limited, the populations compete only for space. Although such ideal conditions are never met in real tumors, the aim of these additional experiments is to demonstrate in a more straightforward manner how the growth and dominance of each phenotype evolve under normoxic conditions and combined with the different vasculature conditions how they depend on intra-tumoral oxygenation levels.

By construction, phenotype 2 is hypoxia-induced invasive. Thus, in the absolute absence of hypoxia, the only difference between the two phenotypes under these ideal conditions is that phenotype 2 is less proliferative than phenotype 1. As can be seen in Figure S5 (Left), phenotype 1 dominates in the population. On the other hand, very rapidly, the growth of phenotype 2 is stalled, while it remains trapped in the tumor core (Figure S5-Right).

1. Oxygen level is kept at maximum up to some point in time throughout tumor evolution

In these set of experiments, we investigate how a tumor consisting of phenotype 1 and phenotype 2 evolves when we delay the onset of hypoxia by keeping the oxygen level at its maximum up to some point in time. After that time, the system evolves under well-vascularized conditions that were described in the main text. In order to speed up the simulations, we decreased the spatial resolution of these experiments so that they were performed on a 200x200 regular grid (as the y-axis is not normalized to the total grid points, a difference in the order of tumor population estimation can be observed i.e. approximately 16 times less). At first, oxygen was kept at its maximum level for the first 40 days of simulation. As can be seen in Figure S6a and S6b, phenotype 2 eventually starts growing. However, if normoxia lasts for the first 100 days of simulation, as can be seen in Figure S6c and S6d, the growth of phenotype 2 is stalled.

Similarly, we investigate how a tumor consisting of phenotype 1 and phenotype 2 evolves when we delay the onset of hypoxia by keeping the oxygen level at its maximum up to some point in time. However, the difference with the previous set of experiments is that after the normoxic period, the system evolves under poor-vascularized conditions. These experiments were performed on a 200x200 regular grid. At first, oxygen was kept at its maximum level for the first 40 days of simulation. As can be seen in Figure S7a and S7b, after a period of time (approximately, 120 days from the beginning of simulations), phenotype 2 starts outgrowing, although the hypoxic sub-populations of both phenotypes are abundant by the end of simulations. However, if normoxia lasts for the first 100 days of simulation, as can be seen in Figure S7c and S7d, the growth of phenotype 2 is stalled. Both set of experiments show that under conditions where the onset of hypoxia substantially delays, phenotype 2 can be trapped in the tumor core where its growth is stalled.

1. Oxygen level is kept at maximum after some point in time throughout tumor evolution

In these set of experiments (Figure S8), we investigate how a tumor consisting of phenotype 1 and phenotype 2 evolves when we reinitialize oxygen at some time point and keep it at its maximum level thereafter. For a given timer period, the tumor evolves under well-vascularized growth conditions and then oxygen re-initialization occurs. In order to speed up the simulations, we decreased the spatial resolution of these experiments so that they were performed on a 200x200 regular grid. As oxygen becomes plentiful, the hypoxic sub-populations turn to normoxic. Interestingly, the outgrowth of phenotype 2 can be stalled when the oxygen re-initialization period begins early enough (Figure S8i).
