# Supplementary material for: Exploring the Competition between Proliferative and Invasive Cancer Phenotypes in a Continuous Spatial Model
Source: PLoS One. 2014 Aug 6;9(8):e103191. doi: 10.1371/journal.pone.0103191 (PMC4123877; doi:10.1371/journal.pone.0103191)
Supplement: Text S2 — Exploring additional changes in the diffusion-proliferation rate map. (DOCX) [file pone.0103191.s020.docx]

**Diffusion rate – Proliferation rate**

Tumor cells are embedded in a fibrous material known as ECM, which is remodeled during tumor growth. The structure and composition of the ECM affect cell adhesion and motility thus, play a critical role in tumor invasion, morphology and metastasis [[1](#_ENREF_1)]. In order to facilitate their movement, tumor cells usually produce matrix degrading enzymes (such as Matrix Metalloproteinases) that degrade the ECM locally. Tumor cells can haptotactically migrate to denser areas of ECM [[1](#_ENREF_1),[2](#_ENREF_2)]. ECM degradation increases the gradient and adds in tumor expansion. In the absence of matrix degradation, cell motility and invasion become more difficult, although other factors that influence ECM remodeling can also affect tumor cell motility.

As described in the main text, in order to keep our model simple, we assumed a random distribution of the ECM ($0\leq f(x,y)\leq1$), which is not degraded or remodeled by any mechanism but remains unchanged throughout tumor growth. Therefore, it is important to mention that when ECM degradation will be included it will probably favor further the invasive phenotypes.

In these set of experiments, we explore the dynamics of tumor populations as we vary the proliferation rate ($\rho_{2}^{*}$) and the diffusion rate ($D_{2}^{*}$) of the invasive phenotype, while keeping the rates of the proliferative, phenotype 1 constant. In addition to the experiments described in the main text where the haptotactic coefficient has been kept the same and equal to *χ* for the invasive phenotype, we also explore the case where no haptotaxis is considered.

As mentioned in the main text, the invasive phenotype can be either conditionally more motile of type of phenotype 2 or unconditionally more motile of type of phenotype 3. We call the phenotype that is constructed when we assign different diffusion and proliferation rates from phenotype 2, *phenotype 2** and *phenotype 3** respectively for alterations of phenotype 3. The co-growth between phenotype 1 and phenotype 2* as well as between phenotype 1 and phenotype 3* are explored.

Figure S16 shows the regions in the parameter space where each phenotype dominates in the presence (top row) and absence (bottom row) of haptotaxis, respectively. Indicatively, the co-growth between phenotype 1 and phenotype 2* were examined under poorly-vascularized conditions (left column) and the co-growth between phenotype 1 and phenotype 3* were examined under well-vascularized conditions (right column). A shift and expansion to the left area of the parameter space can be observed for the invasive phenotypes in the absence of haptotaxis showing that the random ECM in the absence of local matrix degradation makes invasion more difficult.

**References**

1. Zaman MH, Trapani LM, Sieminski AL, Mackellar D, Gong H, et al. (2006) Migration of tumor cells in 3D matrices is governed by matrix stiffness along with cell-matrix adhesion and proteolysis. Proc Natl Acad Sci U S A 103: 10889-10894.

2. Anderson AR (2005) A hybrid mathematical model of solid tumour invasion: the importance of cell adhesion. Math Med Biol 22: 163-186.
